# Rhodium Oxide Surface-Loaded Gas Sensors

**DOI:** 10.3390/nano8110892

**Published:** 2018-11-01

**Authors:** Anna Staerz, Inci Boehme, David Degler, Mounib Bahri, Dmitry E. Doronkin, Anna Zimina, Helena Brinkmann, Sina Herrmann, Benjamin Junker, Ovidiu Ersen, Jan-Dierk Grunwaldt, Udo Weimar, Nicolae Barsan

**Affiliations:** 1 Institute of Physical and Theoretical Chemistry (IPTC), University of Tuebingen, Auf der Morgenstelle 15, D-72076 Tuebingen, Germany; anna.staerz@ipc.uni-tuebingen.de (A.S.); inci.can@ipc.uni-tuebingen.de (I.B.); helena.brinkmann@student.uni-tuebingen.de (H.B.); sina.herrmann@ipc.uni-tuebingen.de (S.H.); benjamin.junker@ipc.uni-tuebingen.de (B.J.); upw@ipc.uni-tuebingen.de (U.W.); 2 European Synchrotron Radiation Facility (ESRF), 71 Avenue des Martyrs, 38043 Grenoble, France; david.degler@esrf.fr; 3 Institut de Physique et Chimie des Matériaux de Strasbourg (IPCMS), UMR 7504 CNRS-Université de Strasbourg, 23 rue du Lœss, F-67034 Strasbourg cedex 2, France; mounib.bahri@ipcms.unistra.fr (M.B.); ovidiu.ersen@ipcms.unistra.fr (O.E.); 4 Institute of Catalysis Research and Technology (IKFT) and Institute for Chemical Technology and Polymer Chemistry (ITCP), Karlsruhe Institute of Technology, Kaiserstr. 12, 76131 Karlsruhe, Germany; dmitry.doronkin@kit.edu (D.E.D.); anna.zimina@kit.edu (A.Z.); grunwaldt@kit.edu (J.-D.G.)

**Keywords:** gas sensors, surface-loading, DRIFT spectroscopy, X-ray absorption spectroscopy, Fermi-level pinning

## Abstract

In order to increase their stability and tune-sensing characteristics, metal oxides are often surface-loaded with noble metals. Although a great deal of empirical work shows that surface-loading with noble metals drastically changes sensing characteristics, little information exists on the mechanism. Here, a systematic study of sensors based on rhodium-loaded WO_3_, SnO_2_, and In_2_O_3_—examined using X-ray diffraction, high-resolution scanning transmission electron microscopy, direct current (DC) resistance measurements, operando diffuse reflectance infrared Fourier transform (DRIFT) spectroscopy, and operando X-ray absorption spectroscopy—is presented. Under normal sensing conditions, the rhodium clusters were oxidized. Significant evidence is provided that, in this case, the sensing is dominated by a Fermi-level pinning mechanism, i.e., the reaction with the target gas takes place on the noble-metal cluster, changing its oxidation state. As a result, the heterojunction between the oxidized rhodium clusters and the base metal oxide was altered and a change in the resistance was detected. Through measurements done in low-oxygen background, it was possible to induce a mechanism switch by reducing the clusters to their metallic state. At this point, there was a significant drop in the overall resistance, and the reaction between the target gas and the base material was again visible. For decades, noble metal loading was used to change the characteristics of metal-oxide-based sensors. The study presented here is an attempt to clarify the mechanism responsible for the change. Generalities are shown between the sensing mechanisms of different supporting materials loaded with rhodium, and sample-specific aspects that must be considered are identified.

## 1. Introduction

As the world becomes more automated and connected, sensors will play an increasing role. Gas sensors based on semiconducting metal oxides (SMOX) are a compact, inexpensive, sensitive, and robust alternative to other detection methods. Over the last five decades, SMOX-based sensors were widely used for automated air flap control in cars and in domestic alarms for explosive gases [1,2]. A great deal of research is being done on the use of SMOX sensors in a wide array of applications. However, in order for SMOX-based sensors to be effectively used in the future, their sensitivity, selectivity, and stability must be increased [3]. Numerous different methods were examined in order to address these issues, ranging from the optimization of morphology to the use of composite materials based on metal oxides coupled with organics or silica [3,4,5]. Traditionally, these limitations were addressed through loading with noble metals [6]. Already in the 1960s, palladium was added to the first commercially available SnO_2_-based sensor from Figaro Engineering [7,8]. Even today, most commercial sensors are not based on pure materials, but contain low quantities of noble-metal-oxide additives [8]. These additives are usually chosen based on empirically attained knowledge. However, in the late 1980s and early 1990s, Yamazoe and Morrison suggested two mechanisms, spillover and Fermi-level pinning, which could explain the effect of surface-loading on the sensor response; experimental results supporting the theories remain limited [9,10,11]. In the case of the spillover mechanism, the target/analyte molecule is adsorbed onto the noble-metal-oxide cluster which leads to a weakening of its molecular bond. The adsorbate is transferred onto the support material where the reaction takes place [12]. In the Fermi-level pinning mechanism, the gas detection reaction takes place on the surface of the noble metal cluster. The cluster electronically interacts with the base material and the contact pins the Fermi levels of both materials. If the work function of the noble-metal-oxide cluster is changed upon interacting with an analyte gas, the depletion layer in the base material caused by the contact is also affected. It was recently reported that the Fermi-level pinning mechanism explains the change in the sensing characteristics of WO_3_ surface-loaded with oxidized rhodium clusters and the effect of oxidized platinum clusters on the surface of SnO_2_ [13]. Here, a systematic study of three commonly used oxides for gas sensors, WO_3_, SnO_2_, and In_2_O_3_ [3], loaded with rhodium oxide clusters was done to examine the general validity of these findings. The response of the sensors to five chemically different and application-relevant gases was examined. The gases were picked in concentrations relevant for different applications. There is currently a large interest in detecting acetone in the breath as a means for diabetes monitoring [14]. For diabetes monitoring, an acetone concentration between 0.5 ppm and 2 ppm (0.001–0.005 mg/L) is relevant [14]. CO and NO_2_ are both relevant pollutants found in automobile exhaust measurements [15]. Values between 30 ppm and 100 ppm (ca. 0.035–0.116 mg/L) are relevant for CO, and between 2 ppm and 10 ppm (0.004–0.019 mg/L) for NO_2_ [16]. Often, however, indoor air is more polluted than outdoors. As people spend more and more time inside, governments and even the World Health Organization released guidelines regarding indoor air quality [17,18]. Indoor air quality is often diminished by the presence of volatile organic compounds due to outgassing of furniture and other household objects. The Canadian government, for example, set a short-term indoor air exposure limit of toluene at 4 ppm (0.015 mg/L) [18]. Ethanol is another volatile organic compound which metal-oxide-based gas sensors are known to respond well to. In total, these gases show high variation in their chemical characteristics and allowing their use in sensors for a wide array of relevant applications.

A full characterization of the samples was done using X-ray diffraction (XRD) and high-resolution scanning transmission electron microscopy (HR-STEM). In order to understand the effect of oxidized noble-metal surface clusters on sensing, operando diffuse reflectance infrared Fourier transform (DRIFT) spectroscopy and operando X-ray absorption spectroscopy (XAS) were used. The results show that the Fermi-level pinning mechanism accurately describes the effect of the clusters on sensing. This work indicates the general validity of the Fermi-level pinning mechanism for sensors based on oxygen-deficient *n*-type SMOX-containing surface noble-metal-oxide clusters. This finding is very significant, as the sensor characteristics of SMOX are often tuned using loading with noble metals.

## 2. Materials and Methods

### 2.1. Sample Preparation

The loaded samples were prepared as described in Reference [19]. SnO_2_/WO_3_/In_2_O_3_ and RhCl_3_∙xH_2_O from Sigma Aldrich (Saint Louis, MI, USA) were stirred in deionized water at a pH value of 1.0 at 80 °C for 2 h and dried at 70 °C. The powders were calcined at 500 °C for 1 h. To ensure that the detected results were caused by the presence of the surface-loading and not due to the preparation procedure itself, the pure samples were also suspended in deionized water at a pH value of 1.0, which was set using a concentrated HCl solution. Then, the suspension was stirred at 80 °C for 2 h and the powders were dried at 70 °C and calcined at 500 °C for 1 h. The powders were deposited onto alumina substrates as described elsewhere [20]. More information regarding the loading can be found in the Table S1.

### 2.2. Direct Current (DC) Resistance Measurements

The measurements were performed using a Keithley 617 electrometer for WO_3_ and SnO_2_ and an Agilent 34972 multimeter for In_2_O_3_. Agilent E3630A and E3614A voltage sources were used to heat the sensors. The sensors were mounted in a homemade Teflon sensor chamber, and the various test gas concentrations in dry synthetic air were supplied using a computer-controlled gas-mixing system. As the standard for work done using gas sensors, the concentration of the target gas is given in ppm. The following relationship was used [21]:

(1) $$\mathrm{Gas}\;\mathrm{Concentration}\;\left(\mathrm{ppm}\right)=\frac{\mathrm{Mole}\;\mathrm{Volume}}{\mathrm{Mole}\;\mathrm{Mass}}\times \mathrm{Gas}\;\mathrm{Concentration}\;\left(\frac{g}{m^{3}}\right)=\frac{{0.241m}^{3}}{\mathrm{Mole}\;\mathrm{Mass}}\times \mathrm{Gas}\;\mathrm{Concentration}\;\left(\frac{g}{m^{3}}\right)$$

The sensors were measured at 300 °C. The sensor signal for reducing gases was calculated using the following Equation (2):

(2) $$s=\frac{R_{\mathrm{reference}}}{R_{\mathrm{test}\;\mathrm{gas}}}$$

In the case of oxidizing gases, the inverse relationship was used. The reference was the resistance measurement in dry synthetic air.

### 2.3. DRIFT Spectroscopy

For the operando DRIFT spectroscopy, a Vertex70v containing a narrow-band mercury cadmium telluride (MCT) detector (Bruker, Billerica, MA, USA) with a spectral resolution of 4 cm^−1^ was used. The sensors were mounted in a homemade chamber containing a KBr window. The sensors were heated to 300 °C, and the DC resistance was recorded simultaneously. Every 15 min, a single-channel spectrum was recorded during the gas exposure. To obtain the absorbance spectra, information about the surface reaction was provided with the target gas. The single-channel spectra taken under exposure to the target gases were referenced to the spectra taken under the carrier gas using Equation (3).

(3) $$\mathrm{Absorbance}=-\log\left(\frac{\mathrm{single}\;\mathrm{channel}\;\mathrm{test}\;\mathrm{gas}}{\mathrm{single}\;\mathrm{channel}\;\mathrm{reference}}\right)$$

As previously reported, it is possible to estimate the band-bending caused by the presence of noble-metal-oxide surface-loadings from these measurements [13,22,23]. In N_2_, the surface acceptor state related to the ionosorption of O_2_ is considered negligible, and the relationship between resistance and surface band-bending (eV_S_) for depletion layer limited charge transport can be estimated using the Equation (4) [13,22,23]:

(4) $${\mathrm{eV}}_{s}=\mathrm{kT}\cdot \ln\left(\frac{R_{\mathrm{loaded}}}{R_{\mathrm{pure}}}\right)$$

where k represents the Boltzmann constant, T is the temperature, R_pure_ is the resistance of the unloaded material, and R_loaded_ is the resistance of the loaded material in N_2_.

### 2.4. XRD Measurements

XRD diffractograms were collected with a Philips X’Pert apparatus (PANalytical Spectris, Egham, UK). A monochromatic Cu Kα radiation source (λ = 1.540598 Å) was used. The diffractograms were recorded from a 2θ-ω angle of 25° to 45° with a step size of 0.01° at a rate of 0.01°/s. The XRD data were analyzed using the Match! 3 software (CRYSTAL IMPACT, Bonn, Germany).

### 2.5. X-ray Absorption Spectroscopy (XAS) Measurements

All X-ray absorption spectroscopy (XAS) experiments were recorded at beamline P65 at the PETRA III synchrotron radiation source (DESY, Hamburg, Germany). X-rays were provided by an undulator (11 periods, seventh harmonic, DESY, Hamburg, Germany); higher harmonics were rejected using Pt-coated mirror layers mounted before the monochromator, and the incident X-ray energy was selected using a double crystal monochromator with Si (311) crystals. Using slits, the beam size was set to 1.5 × 0.3 mm. X-ray absorption near-edge structure (XANES) and extended X-ray absorption fine structure (EXAFS) spectra were recorded at the Rh K-edge in fluorescence geometry using an energy-dispersive Vortex P80 detector. For operando XAS, the samples were placed in a homemade in situ cell, which allowed controlling the atmosphere and heating voltage, while simultaneously recording XAS and DC resistance of the sensor placed in the cell [24]. XAS data analysis was done using the ATHENA and ARTEMIS software form the IFEFFIT package (developed by Ravel and Newville from the US Naval Research Laboratory and the University of Chicago, for details see [25]). ATHENA was used for calibrating and normalizing all spectra, and for subtracting the background of the EXAFS. Fourier transformation (FT) of k^1^, k^2^, and k^3^-weighted EXAFS was done in a k range of 2.5 Å^−1^ to 10 Å^−1^, using a Hanning window with a sill size of 1 Å^−1^. Using ARTEMIS, EXFAS fitting was done by adjusting theoretical backscattering paths, which were obtained from FEFF 6.0 calculations (The FEFF Project, University of Washington, WA, USA, to experimental data using the least-squares method in R space (1 Å to 3.5 Å) [26].

### 2.6. Scanning Transmission Electron Microscopy

Scanning transmission electron microscopy (STEM) experiments were performed using a Cs-corrected JEM-2100F (JEOL Akishima, Tokyo, Japan) operated at 200 kV.

## 3. Results

### 3.1. Material Characterization

WO_3_, SnO_2_, and In_2_O_3_ are all oxygen-deficient *n*-type semiconductors that are commonly used for gas sensors. The sensors are typically operated between 200 °C and 500 °C. Here, in order to minimize the measurement variables, an operation temperature of 300 °C was selected.

**Figure 1:**
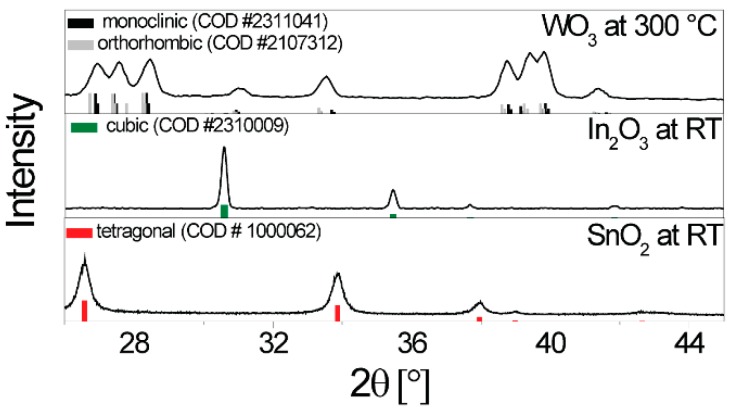
X-ray diffractograms taken of the sensitive layer on the sensors (SnO_2_ and In_2_O_3_ at room temperature; WO_3_ was recorded on a heated sensor). The reference patterns are from the Crystallography Open Database.

XRD measurements were taken at room temperature for SnO_2_ and In_2_O_3_, while that of WO_3_ was taken at 300 °C (see Figure 1). For SnO_2_, the tetragonal rutile structure was verified [27], and, as it only has one stable crystal structure, it can be assumed that the same structure exists at the operation temperature. As already reported in the case of WO_3_, the sample is found to be in a mixed γ- and β-phase at 300 °C [28,29,30]. For In_2_O_3_, the cubic structure was verified by XRD [31]. This structure is stable until 800 °C, which is well above the operation temperature; thus, no change was anticipated [32]. Using the Debye-Scherrer equation, the crystallite diameters of the base materials were approximated as 30 nm for WO_3_, 20 nm for SnO_2_, and 40 nm for In_2_O_3_.

**Figure 2:**
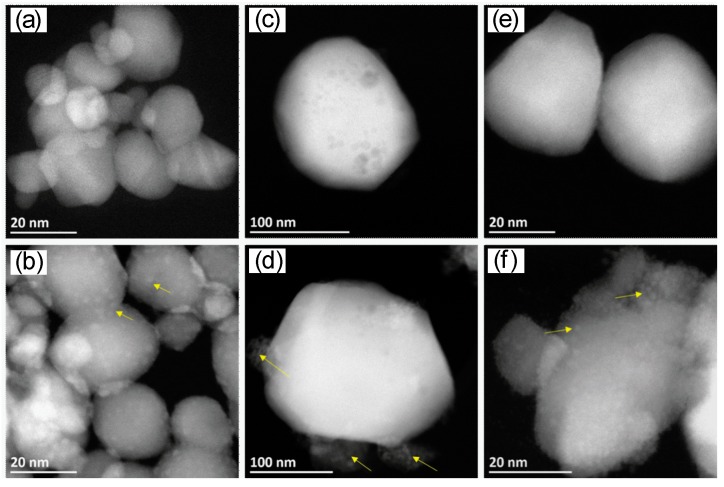
Scanning transmission electron microscopy (STEM) high-angle annular dark-field (HAADF) images of pure and Rh-loaded clusters on three different supports: SnO_2_ (**a**) pure and (**b**) 3.00 at.% Rh-loaded SnO_2_; In_2_O_3_ (**c**) pure and (**d**) 2.75 at.% Rh-loaded In_2_O_3_; WO_3_ (**e**) pure and (**f**) 5.00 at.% Rh-loaded WO_3_. Yellow arrows show the Rh_2_O_3_ particles and clusters.

In HR-STEM images, however, it can be seen that the In_2_O_3_ clusters were larger than 100 nm, meaning that the crystallite size could not be accurately determined using the Debye-Scherrer equation [33]. The average size of the In_2_O_3_ clusters was approximately 300 nm. In the case of the SnO_2_ sample, the grain size was between 7 nm and 25 nm. In the case of the WO_3_ sample, the crystallite size varied between 25 nm and 65 nm. These findings are in line with the XRD measurements. The Rh-oxide loading is marked with yellow arrows in Figure 2. The STEM measurements revealed that, for loaded WO_3_ and SnO_2_, the rhodium-oxide particle size was between 1 nm and 2.5 nm with a good dispersion on the surface (Figure 2).

**Figure 3:**
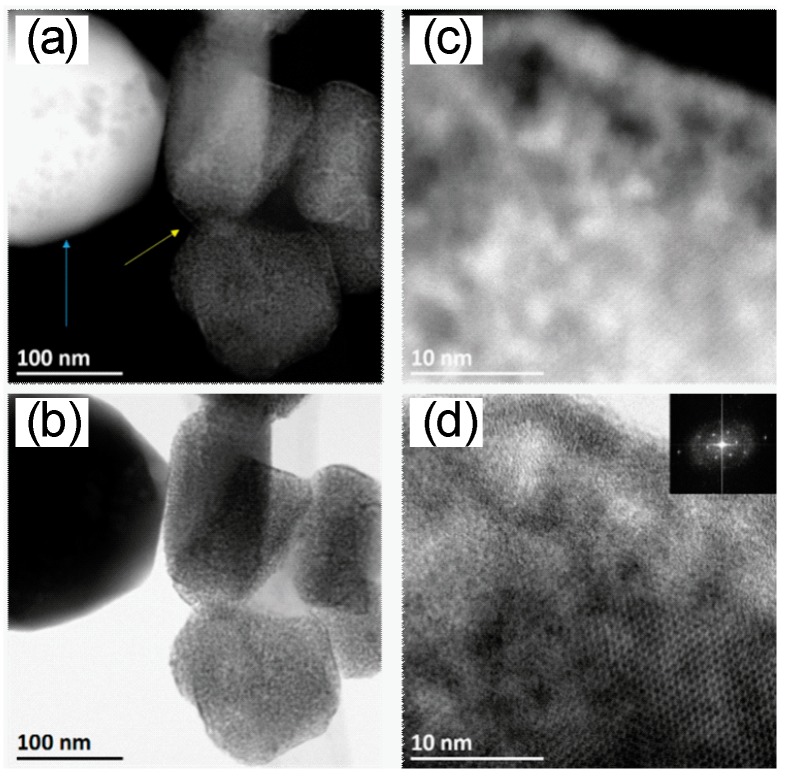
STEM images of 2.75 at.% Rh-Loaded In_2_O_3_. (**a**) STEM HAADF and (**b**) STEM bright-field (BF) images show two kinds of structures: low Rh-loaded (blue arrows) and high Rh-loaded (yellow arrow). (**c**) High-resolution (HR)-STEM HAADF and (**d**) HR-STEM BF images show high crystalline In_2_O_3_ and a rich Rh layer around In_2_O_3_. The inset in (**d**) is the fast Fourier transform (FFT) of the STEM BF image.

For loaded In_2_O_3_, however, there were two kinds of structures: one with a high amount of Rh_2_O_3_ on a small amount of In_2_O_3_ (yellow arrow, Figure 3a), and the second with large crystals of In_2_O_3_ and a small quantity of rhodium oxides (Figure 2d; blue arrow Figure 3a; barely visible in Figure 3b). The high amount of Rh_2_O_3_ is shown as a layer formed around a highly crystalline small In_2_O_3_ grain (Figure 3c,d). Energy-dispersive X-ray spectroscopy (EDS) elemental mapping images support these findings (see Figure S1).

**Figure 4:**
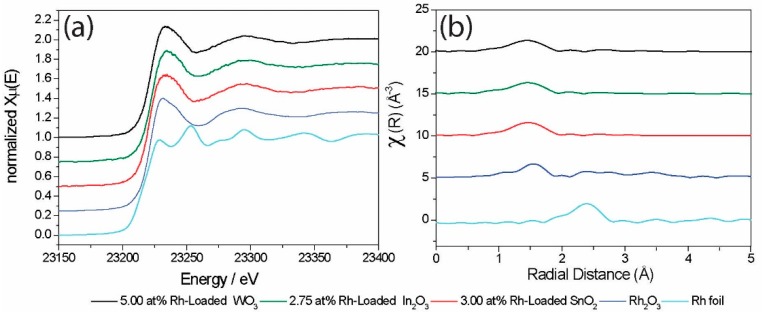
Rh K-edge X-ray absorption near-edge structure (XANES) spectra (**a**) and Fourier transform (FT) k^3^-weigthed extended X-ray absorption fine structure (EXAFS) spectra (**b**) of Rh-loaded sensing materials (amplitude×2), Rh_2_O_3_ reference (amplitude×2), and Rh foil $\left(\frac{\mathrm{amplitude}}{2}\right)$.

The XANES spectra, at room temperature, of all sensing materials and two reference compounds are shown in Figure 4. All three sensing materials showed similar XANES spectra which corresponded to the Rh_2_O_3_ reference compound, i.e., Rh was present as Rh^3+^. Further information on the structure of the Rh loadings was gained through analysis of the EXAFS. The visual inspection of the FT EXAFS (Figure 4b) shows good agreement with Rh_2_O_3_ in the first coordination shell and, thus, confirms the presence of oxidized Rh structures. The strongly decreased magnitude of features related to the outer shells suggests a small size of the Rh loadings. Quantitative information on the Rh structure was obtained by fitting the calculated EXAFS based on Rh_2_O_3_ to the experimentally obtained EXAFS. The results of the best fits are shown in Table 1.

For the 5.0 at.% Rh-loaded WO_3_ sample, the best EXAFS fit was obtained by a model with two coordination shells. The first shell consisted of six O atoms at a distance of 2.02 Å, while the second shell featured two Rh atoms at a distance of 3.09 Å. The coordination of Rh by six O atoms corresponds to the theoretical coordination number. The fitted Rh–O distance was shorter than expected for bulk Rh_2_O_3_ (2.04 Å), but still closer to that of Rh_2_O_3_ than the average W–O distance of the supporting WO_3_ (1.93 Å). The coordination number of the second shell was smaller than the theoretical value of 3, and the obtained Rh–Rh distance was longer than the expected value of the corresponding shell in bulk Rh_2_O_3_ (2.99 Å). The introduction of additional Rh shells found for bulk Rh_2_O_3_ at either at 2.72 Å (N of bulk Rh_2_O_3_: 1.0) or 3.58 Å (N of bulk Rh_2_O_3_: 3.0) did not improve the fit model.

For the 3.0 at.% Rh-loaded SnO_2_ and the 2.75 at.% Rh-loaded In_2_O_3_, the best fits were obtained by a model with three coordination shells. In both cases, the best fits were obtained by adjusting the coordination number of oxygen (first shell) to 5.5, which was smaller than the theoretical value of 6.0, but still matched an octahedral coordination of Rh by O. The fitted Rh–O distance (2.04 Å) was similar for both materials and corresponded to that of bulk Rh_2_O_3_. The second and third coordination shells were fitted by Rh atoms at distances of 3.14 and 3.34 Å for 3.0 at.% Rh-loaded SnO_2_, and 3.15 and 3.38 Å for 2.75 at.% Rh-loaded In_2_O_3_. Considering the calculated errors, the second and third shells were within similar distances for both materials. The used coordination numbers (Table 1) were smaller than the theoretically expected value of 3.0 for each shell in bulk Rh_2_O_3_. The Rh-loaded In_2_O_3_ sample was fitted with significantly lower coordination numbers than the Rh-loaded SnO_2_ sample.

All three materials were successfully fitted with fit models derived from Rh_2_O_3_. Thus, an incorporation of Rh into the lattice of the supporting oxide could be excluded. Rh-loaded SnO_2_ and In_2_O_3_ showed a similar Rh_2_O_3_-like structure, and the decreased coordination numbers and high disorder of the second and third shells suggested a small size of Rh_2_O_3_ clusters on SnO_2_ and In_2_O_3_. The coordination numbers of the second and third shells of an oxide decrease with decreasing particle size, e.g., as reported for NiO [34]. Thus, based on the coordination number, the Rh_2_O_3_ clusters on SnO_2_ were expected to be larger than those on In_2_O_3_. For Rh-loaded WO_3_, the quantitative analysis of the EXAFS showed a less Rh_2_O_3_-like structure. However, based on the Rh–O distance, which was still close to that of Rh_2_O_3_, and based on the presence Rh in the second coordination shell, the structure was assumed to still be Rh_2_O_3_-like. The shortened Rh–O distance, high disorder, low coordination number of the second shell, and atypical Rh–Rh distance suggested the presence of very small and highly disordered Rh_2_O_3_-like clusters on WO_3_.

### 3.2. Resistance Measurements

**Figure 5:**
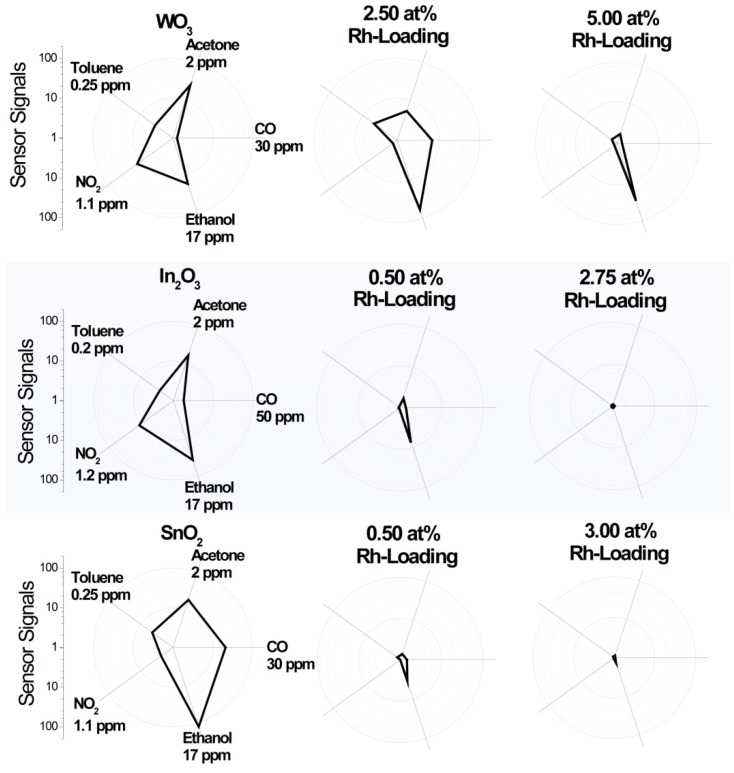
Signals of the sensors based on the different materials with various test gases in dry air at an operation temperature of 300 °C. The results are shown in a polar plot in order to highlight the general qualities of the sensors.

**Figure 6:**
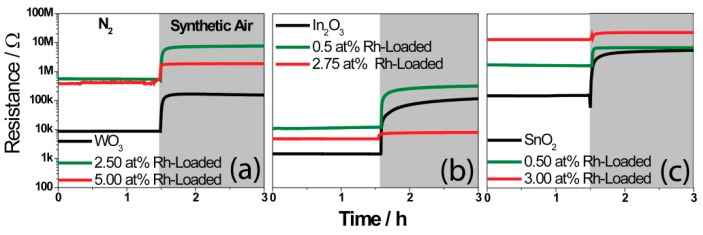
The resistances of the sensors, based on WO_3_ (**a**), In_2_O_3_ (**b**), and SnO_2_ (**c**), are shown in N_2_ and dry synthetic air background (80% N_2_ and 20% O_2_).

The goal of the study was to understand how the presence of the oxidized noble-metal clusters changes sensing. In order to identify changes in the sensor characteristics, sensors based on the pure base material must show significant and stable responses. Although it is known that sensors based on materials which are highly loaded with noble metals show responses at lower temperatures, the sensors based on pure base materials need a higher operation temperature. For this reason, the DC resistance measurements were conducted at 300 °C (see Figure 5 and Figure 6). The sensor signals were calculated using Equation (2), and the results are shown in Figure 5. Although, for real world applications, the effect of humidity must be considered, the work here sought to understand the fundamental mechanism responsible for the change in sensor response. Thus, for the sake of simplicity, the sensor response measurements were done in dry air. In all three cases, the loading resulted in a drastic change of the sensing characteristics. It was previously reported in the 1990s, by Buedy et al., that loading with rhodium drastically changes the sensor response of SnO_2_ [35]. The change in the sensing mechanism, as a result of loading WO_3_ with Rh_2_O_3_ nanoclusters, was recently reported [13]. In this work, a surface-loading of 2.50 at.% was used, and, to corroborate the previous results, a similar loading level was selected here. Based on the crystallite size calculated from the XRD spectra, loadings of 2.75 at.% for In_2_O_3_ and 3.00 at.% for SnO_2_ were selected in order to attain comparable surface-loadings. In both cases, however, the effect of the loading was more significant (negligible sensor responses to all gases) than for the WO_3_ sample [36,37]. For these cases, a lower loading of 0.50 at.% was then additionally examined, while, for WO_3_, a higher loading of 5 at.% was added. In the case of In_2_O_3_, the total surface area was much lower than expected from the XRD measurements (Figure 1), and from the large grains visible in the STEM images (Figure 2). This is a possible explanation for the stronger effect of loading on In_2_O_3_ (comparable results at much lower concentrations) in comparison to WO_3_. Due to the large crystallite size of In_2_O_3_, the ratio of surface area to volume was much smaller than for SnO_2_ and WO_3_ (ca. 0.02 for In_2_O_3_, 0.22 for SnO_2_, and 0.20 for WO_3_). In the case of SnO_2_, there was no identifiable microstructural reason for the stronger effect of the loading. This indicates that the different electronic properties of the base materials also play a role. The results for 2.50 at.% loaded WO_3_ are similar to those previously reported on a differently prepared sample [13]. The 2.50 at.% loading led to the disappearance of the NO_2_ response. Rh_2_O_3_ is a known catalyst for the oxidation of NO to NO_2_, indicating it would be a poor sensing material for NO_2_ [38]. The response to CO increased; CO is known to react with the lattice oxygen of Rh_2_O_3_ to form CO_2_ [39]. The response to acetone decreased, and there was practically no change in the response to toluene. Here, it was additionally found that the response to ethanol increased. For sensors based on the 5.00 at.% loaded WO_3_ sample, the response to all gases except ethanol became negligible.

A very significant change in the sensing characteristics of In_2_O_3_ can already be seen for the 0.50 at.% loaded sample. Like WO_3_, In_2_O_3_ is known to respond well to NO_2_. This inherent characteristic of In_2_O_3_ disappeared as a result of the loading. Similar to the loaded WO_3_ sample, the response to acetone decreased. The response to CO remained practically unchanged. The response of the 2.75 at.% to all gases was indeterminable. The effect of 0.50 at.% loading also had a significant effect on SnO_2_. The sensor response was very similar to that of the 0.50 at.% loaded In_2_O_3_ sensor. The response of the 3 at.% loaded SnO_2_ sample was negligible for all gases.

In order to examine the electronic coupling between the oxidized rhodium clusters and the host oxide, resistance measurements were conducted in N_2_.

The large increase of the resistance in nitrogen as a result of the loading indicates a strong electronic coupling between the surface clusters and the base material. The heterojunction resulted in a depletion layer which extended into the *n*-type base material, resulting in a higher resistance. Using the resistance values in nitrogen (Figure 6) of the pure base materials and of the loaded materials, it is possible to calculate the band-bending caused by the noble-metal surface clusters [13,22]. The calculated band-bending of the Rh_2_O_3_ loaded materials are shown in Table 2. This was done using Equation (4).

In the presence of synthetic air, the resistance increased due to the adsorption of the oxygen. The pure materials all showed large changes in resistance (Figure 6). The effect of oxygen was lower for the loaded samples. While the oxidation of the Rh-clusters by atmospheric oxygen (dominant effect on loaded samples) would also lead to an increase in the resistance, it appears to be less effective than the direct oxidation of the base material (effect of atmospheric oxygen on the pure materials). Interestingly, for the WO_3_ sample, the band-bending caused by the surface clusters was similar for the 2.50 at.% and the 5 at.% loaded samples. The effect of oxygen was, however, much more significant for the 2.50 at.% loaded sample [36]. One possible explanation is that the dispersion of the clusters was similar for the two samples, but the clusters on the 5.00 at.% sample were significantly larger. A similar situation was most probably true for In_2_O_3_. In the case of SnO_2_, the calculated band-bending was significantly smaller for the lower loading versus the higher sample. The effect of atmospheric oxygen was also, however, lower for the higher-loaded sample. This could indicate that the clusters were larger but also more homogeneously dispersed in the case of the 3.00 at.% loaded sample. When comparing the results of the nitrogen and synthetic air measurements with the sensor response, it becomes clear that the reactivity to oxygen is correlated with the strength of the response to the test gases.

### 3.3. DRIFT Measurements

**Figure 7:**
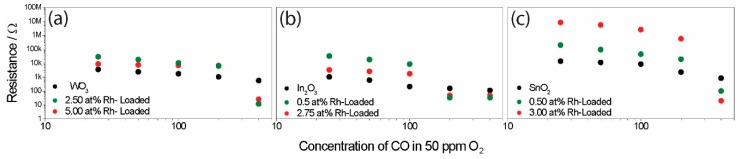
The change in resistance of the sensors, based on WO_3_ (**a**), In_2_O_3_ (**b**), and SnO_2_ (**c**), during exposure to different CO concentrations in a low-oxygen background, measured during the simultaneously measured diffuse reflectance infrared Fourier transform (DRIFT) spectroscopy, is plotted in log–log.

Following surface-loading with oxidized rhodium clusters, the responses of the different base materials became similar (see Figure 5). In order to examine how the presence of the surface additives unified the sensing characteristics, the interaction between the sensors and CO, an exemplary gas, was examined in greater detail. To examine the surface reactions, operando DRIFT spectroscopy was used. This method was proven to be a powerful tool [40]. In order to examine the role of the oxidized rhodium clusters in sensing and to induce a mechanism switch by reducing the clusters to their metallic state, the samples were exposed to different CO concentrations (between 25 ppm and 400 ppm) in a low-oxygen background (50 ppm O_2_). The simultaneously obtained electrical measurements are shown in Figure 7.

**Figure 8:**
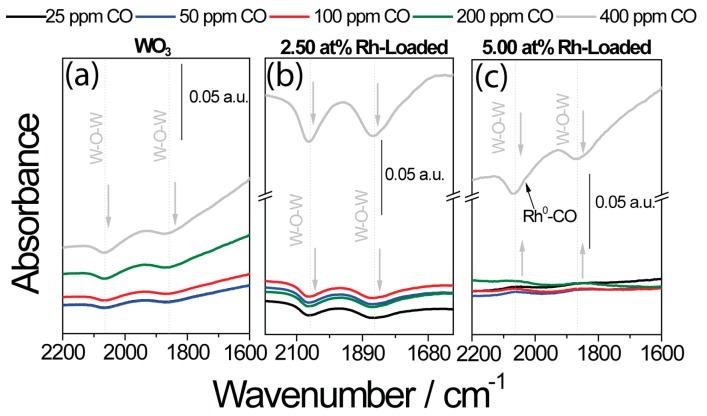
DRIFT spectra taken during exposure to different CO concentrations in low-oxygen (50 ppm O_2_) backgrounds for (**a**) unloaded WO_3_ sample, (**b**) 2.50 at.% Rh_2_O_3_ loaded WO_3_, and (**c**) 5 at.% Rh_2_O_3_ loaded WO_3_.

As expected, the sensor signals for the unloaded samples initially increased with higher concentrations, and, at higher concentrations, the sensitivity (the change in sensor response per ppm) decreased as a result of saturation (Figure 7). In the case of the loaded sample, there was a significant jump in the sensor response at higher CO concentrations (in the cases of WO_3_ and SnO_2_, this jump occurred between 200 ppm and 400 ppm, while, for indium, the jump occurred between 100 ppm and 200 ppm). The simultaneously acquired DRIFT spectra also showed a much higher absorbance at this point (Figure 8). It is known that the free-carrier absorption is proportional to the density of conduction electrons. For this reason, the resistance is inversely proportional to the absorbance, explaining the large change in the overall absorbance in the spectra taken under high CO concentrations [41]. In the 1960s, this phenomenon was examined in detail by Harrick for an oxidized silicon surface [42]. In addition, discrete adsorption bands in the DRIFT spectra provide information about the surface reactions responsible for the sensing at this point. In the case of pure WO_3_ (Figure 8a), the exposure to CO determined the decrease of the W–O lattice bands at 2061 cm^−1^ and 1853 cm^−1^ [43]. This indicates the reduction of the material’s surface.

The sensing mechanism was different for 5 at.% loaded WO_3_ (Figure 8c). Here, an increase of the W–O bands was visible for low CO concentrations. This finding was previously reported for Rh-loaded WO_3_ during exposure to reducing gases such as acetone under normal sensing conditions [13]. As a result of the surface Rh_2_O_3_ clusters, the surface of WO_3_ is highly depleted (see Table 2). As a result of the reaction between CO and the Rh_2_O_3_ clusters, electrons are released back into WO_3_. With these electrons, WO_3_ can react with atmospheric oxygen. This oxidation would, in turn, result in an increase in resistance. The mechanism changed as a result of exposure to 400 ppm, resulting in only the reduction of WO_3_ being visible. This mechanism change was again correlated with the significant decrease in the resistance seen in the electrical measurements. In the DRIFT spectra of the 2.50 at.% loaded WO_3_ sample, the reduction of WO_3_ was visible even at low CO concentrations. Once the Rh_2_O_3_ cluster was reduced, however, CO reacted entirely with the base oxide, and the intensity of the decreasing bands attributed to the reduction of the WO_3_ lattice was significantly heightened. This correlates with the stronger decrease in resistance detected in the electrical measurements.

**Figure 9:**
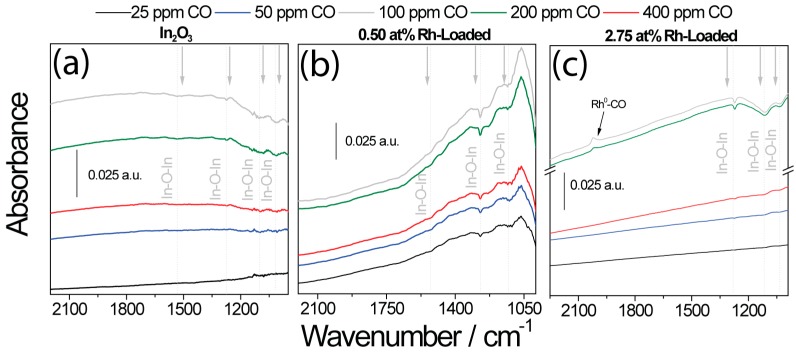
DRIFT spectra taken during exposure to different CO concentrations in low-oxygen (50 ppm O_2_) backgrounds for (**a**) unloaded In_2_O_3_ sample, (**b**) 0.50 at.% Rh_2_O_3_ loaded In_2_O_3_, and (**c**) 2.75 at.% Rh_2_O_3_ loaded In_2_O_3_.

The DRIFT spectra taken of the pure and loaded In_2_O_3_ samples showed a similar situation (Figure 9). The simultaneously acquired DRIFT spectra of pure In_2_O_3_ showed the reduction in the number of In–O bands centered at 1517 cm^−1^, 1274 cm^−1^, 1108 cm^−1^, and 1022 cm^−1^, which indicates the reduction of the surface by CO [37,44]. In the case of the highly loaded sample, the decrease in the number of In–O bands appeared only in higher CO concentrations (200 ppm and 400 ppm CO) [45]. In these concentrations, the jump in the sensor signal appeared. There was also an additional band centered at 2028 cm^−1^, which was assigned to metallic rhodium carbonyls (Rh–CO) [37,44,46].

The DRIFT spectra of the loaded SnO_2_ samples were more difficult to interpret, but showed a similar situation.

**Figure 10:**
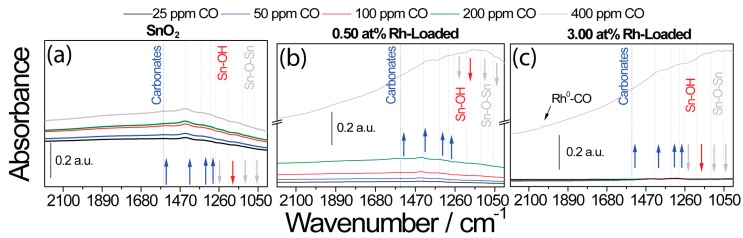
DRIFT spectra taken during exposure to different CO concentrations in low-oxygen (50 ppm O_2_) backgrounds for (**a**) unloaded SnO_2_ sample, (**b**) 0.50 at.% Rh_2_O_3_ loaded SnO_2_, and (**c**) 3.00 at.% Rh_2_O_3_ loaded SnO_2_.

The DRIFT spectra of the sensors based on the SnO_2_ samples are shown in Figure 10. It was previously reported that the reduction of the SnO_2_ lattice by CO is the initial step of the surface reaction [47]. The formed CO_2_ subsequently reacts with the SnO_2_ surface to form carbonates. The DRIFT spectra taken of the pure SnO_2_ sample were in line with this mechanism (Figure 10a). There were decreasing bands visible at 1060 cm^−1^ [48], 1120 cm^−1^ [48], and 1270 cm^−1^ [47], which were attributed to the lattice oxygen of SnO_2_. The increasing bands at 1302 cm^−1^, 1348 cm^−1^, 1442 cm^−1^, and 1561 cm^−1^ were attributed to surface carbonates [49]. In the case of the highly loaded sample (Figure 10c), there was no visible reduction of SnO_2_ or carbonate formation during exposure to low CO concentrations.

In total, the DRIFT spectra provided good insight into how the presence of the Rh_2_O_3_ nanoclusters changed sensing. All three base oxides were reduced by the presence of CO in low-oxygen backgrounds. As expected, the sensitivity of the materials decreased with higher CO concentrations.

In the case of the highly loaded samples, the reduction of the base material only took place in high CO concentrations. This indicates that the reaction mechanism for each material was different at low CO concentration from that of the pure material.

### 3.4. X-ray Absorption Spectroscopy (XAS)

The DRIFT spectra predominantly provided insight into how the interaction between the base oxides and CO changed as a result of loading. In order to gain insight into how the Rh_2_O_3_ clusters changed during sensing, operando XAS was used. Here, the sensors were again exposed to CO (between 25 ppm and 400 ppm CO) in low-oxygen (50 ppm) concentrations. XAS is an element-selective technique, and the shape of the XANES spectra is sensitive to the oxidation state and chemical surrounding of the absorbing element [50,51]. In order to study the behavior of Rh, the same measurements investigated by operando DRIFT spectroscopy of the higher-loaded samples were repeated, simultaneously recording XANES spectra at the Rh K-edge and the sensor response.

**Figure 11:**
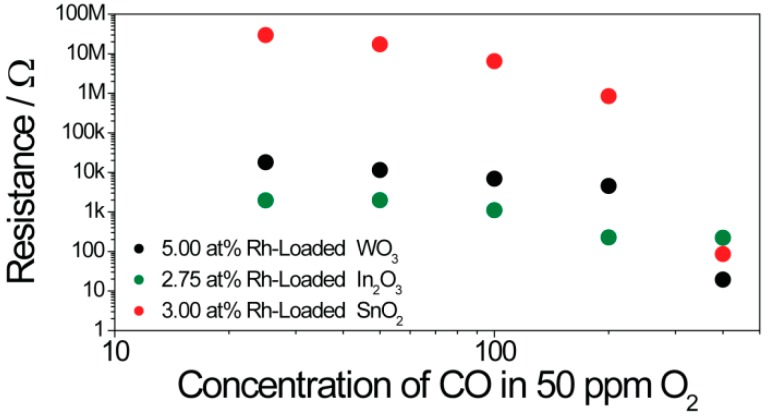
The change in resistance of the sensors during exposure to different CO concentrations in low-oxygen background, acquired during simultaneously measured X-ray absorption spectroscopy (XAS), is plotted in log-log.

The electrical response (Figure 11) of the sensors was very similar to that attained during the DRIFT spectroscopy measurements (Figure 7). The loaded WO_3_ and SnO_2_ samples showed a significant resistance jump under exposure to 400 ppm CO. In the case of In_2_O_3_, the resistance jump was already visible during exposure to 200 ppm CO.

**Figure 12:**
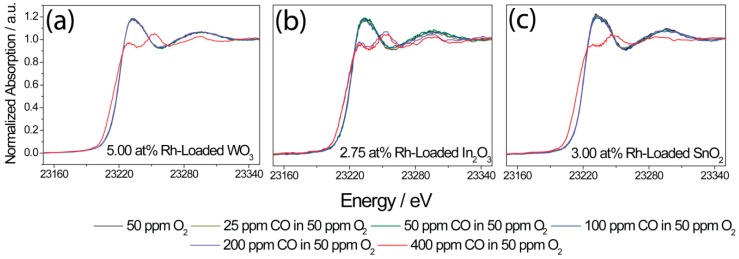
Rh K-edge XANES spectra of 5.00 at.% Rh-loaded WO_3_ (**a**), 2.75 at.% Rh-loaded In_2_O_3_ (**b**), and 3.00 at.% Rh-loaded SnO_2_ (**c**), recorded during different CO exposures in a low-oxygen background (50 ppm O_2_) at 300 °C.

For Rh-loaded WO_3_ and SnO_2_, the XANES spectra showed a change from Rh^3+^ to Rh^0^ between 200 ppm and 400 ppm CO, and, for Rh-loaded In_2_O_3_, the change was already visible between 100 ppm and 200 ppm CO (Figure 12). The oxidation state change of Rh agreed well with the resistance jump and the distinct changes in the DRIFT spectra. Prior to the resistance jump, no indications of metallic Rh were visible in the XANES spectra, i.e., metallic Rh was only found in strongly reducing conditions, while, in oxygen-rich conditions, Rh was oxidized (Rh^3+^). Here, it is important to note that, once the rhodium clusters were completely reduced to their metallic state, the sensors were irreversibly changed, i.e., the resistance in dry air did not return to the same value, and the color of the sensitive layer was different compared to that of a new sensor. In the case of normal operation conditions, the clusters were not reduced to their metallic state, and the sensor response showed good recovery (see XANES spectra recorded during exposure to CO in dry and humid synthetic air; Figure S2).

## 4. Discussion and Conclusion

It was found that, through surface-loading with oxidized rhodium clusters, it is possible to drastically change the sensing characteristics of WO_3_, In_2_O_3_ and SnO_2_. The three metal oxides, which are very commonly used for gas sensors, show very different responses in their pure states. The surface-loading with Rh resulted in more unified responses; none of the sensors responded to NO_2_, while the response of all sensors to acetone decreased, and, at lower loadings, the response to CO was detectable. In all cases, the rhodium-loaded samples showed the highest responses to ethanol. It was previously reported that the Fermi-level pinning mechanism explains the change in the sensing characteristics of WO_3_ surface-loaded with oxidized rhodium clusters, and also the effect of oxidized platinum clusters on the surface of SnO_2_ [13].

In order to generally validate these findings, it was examined whether the change in sensor response of the three most commonly used materials as a result of Rh_2_O_3_ loading could be explained using the Fermi-level pinning mechanism. In all cases, the oxidized rhodium surface clusters caused a significant increase in the material’s resistance in nitrogen, indicating significant electronic coupling between the clusters and the base material. By increasing the loading concentration, the effect of oxygen on the resistance of the materials was lowered and, in all cases, the response of the sensors decreased; this can be explained by the increase in the size of the Rh_2_O_3_ nanoclusters, limiting the charge transfer between the cluster and the base material during the reaction with the target gas. Using measurements in low-oxygen backgrounds, it was possible to identify the effect of the oxidation state of rhodium on the sensing mechanism. The XANES measurements revealed that, under normal sensing conditions, the surface rhodium clusters were oxidized. This was also true for measurements conducted at low CO concentrations in low-oxygen backgrounds. At higher CO concentrations, our results indicate that the clusters were reduced, and only metallic rhodium was present on the surface. A great deal of work was previously done on the interaction between rhodium and CO in catalysis [52,53]. The findings here are in line with those of Basini et al., who found that, at or above 300 °C, for rhodium clusters on various support oxides, only linear or bridged carbonyls were visible, indicative of Rh^0^ [54]. In operando DRIFT spectra taken under the same conditions, the reduction of the base material could only be seen once the surface rhodium clusters were metallic. In the case of WO_3_, the oxidation of the material was visible even in conditions where the rhodium clusters were still oxidized. This indicates that, in normal sensing conditions, when the rhodium clusters are oxidized (Figure S2), reactions between the rhodium clusters and the target gases are responsible for the sensor response. These findings are significant for the intentional tuning of sensors characteristics; the surface chemistry, and, as a result, the sensing characteristics of the sensor are dominated by the noble-metal loading. Although this was true for the three base materials, different loading levels were needed to attain similar results. This indicates that the base material still plays a role in the sensing mechanism. In order to verify this finding and examine it more closely, in the future, base materials with comparable crystallite size and morphology are needed. Hence, it is crucial that a preparation method is developed in which the Rh_2_O_3_ surface cluster size and dispersion are homogeneous among the different samples.

The work presented here provides significant evidence for the general validity of the Fermi-level pinning mechanism for sensors based on Rh_2_O_3_-loaded *n*-type oxides. For decades, sensors based on oxides loaded with noble metals were used in numerous applications. Although theories were developed to explain the changes in sensing characteristics as a result of the loading, experimental evidence is still scarce. The comprehensive study presented here is a crucial first step in understanding the effect of surface-loading. It identifies generalities between different supporting materials, as well as the additional sample-specific aspects that must be considered.

## Figures and Tables

**Table 1:** Structural parameters obtained from fitted extended X-ray absorption fine structure (EXAFS). Coordination number, N; distance, R; Debye–Waller factor, σ^2^; energy shift, δE_0_; passive electron reduction factor, S_0_^2^; misfit, ρ.

Sample	Atom	N	R	σ^2^	S_0_^2^	δE_0_	ρ
			(Å)	(10^−3^ Å^2^)		(eV)	(%)
5.0 at.% Rh WO_3_	O	6.0 ^f^	2.02 ± 0.01	4.01 ± 1.75	0.72 ± 0.09	−2.10 ± 1.14	1.7
	Rh	2.0 ^f^	3.09 ± 0.02	5.25 ± 2.52			
3.0 at.% Rh SnO_2_	O	5.5 ^f^	2.04 ± 0.01	3.22 ± 1.38	0.84 ± 0.08	−0.65 ± 0.94	1.1
	Rh	2.0 ^f^	3.14 ± 0.03	4.39 ± 3.75			
	Rh	2.5 ^f^	3.34 ± 0.03				
2.75 at.% Rh In_2_O_3_	O	5.5 ^f^	2.04 ± 0.01	3.45 ± 1.18	0.72 ± 0.06	−1.14 ± 0.86	0.9
	Rh	1.5 ^f^	3.15 ± 0.03	5.95 ± 4.41			
	Rh	1.5 ^f^	3.38 ± 0.04				

^f^ fixed.

**Table 2:** A list of the calculated band-bending values.

Material	Band-bending (meV)
2.50 at.% Rh-Loaded WO_3_	ca. 198
5.00 at.% Rh-Loaded WO_3_	ca. 200
0.50 at.% Rh-Loaded In_2_O_3_	ca. 105
2.75 at.% Rh-Loaded In_2_O_3_	ca. 98
0.50 at.% Rh-Loaded SnO_2_	ca. 119
3.00 at.% Rh-Loaded SnO_2_	ca. 222

## Supplementary Materials

The following are available online at http://www.mdpi.com/2079-4991/8/11/892/s1, Table S1: Concentrations used for the loading preparations, Figure S1: STEM images of 2.75 at.% Rh-Loaded In_2_O_3_. (a) STEM-HAADF (b) STEM-BF and EDS elemental mapping images (c), STEM images of 5 at.% Rh-Loaded WO_3_. (d) STEM-HAADF (e) STEM-BF and EDS elemental mapping images (f), Figure S2: Rh K-edge XANES spectra of 5 at.% Rh-loaded WO_3_ (a), 2.75 at.% Rh-loaded In_2_O_3_ (b) and 3.00 at.% Rh-loaded SnO_2_ (c), recorded during different CO exposure in dry syn. air at 300 °C.
