# Supplementary material for: Rhodium Oxide Surface-Loaded Gas Sensors
Source: Nanomaterials (Basel). 2018 Nov 1;8(11):892. doi: 10.3390/nano8110892 (PMC6266552; doi:10.3390/nano8110892)
Supplement: Supplementary file 1 [file nanomaterials-08-00892-s001.zip › nanomaterials-375419-supplementary.docx]

**Supporting Information**

Rhodium Oxide Surface-Loaded Gas Sensors

Anna Staerz ^1^, Inci Boehme ^1^, David Degler ^2^, Mounib Bahri ^3^, Dmitry E. Doronkin ^4^, Anna Zimina ^4^, Helena Brinkmann ^1^, Sina Herrmann ^1^, Benjamin Junker ^1^, Ovidiu Ersen ^3^, Jan-Dierk Grunwaldt ^4^, Udo Weimar ^1^ and Nicolae Barsan ^1,^*

^1^ Institute of Physical and Theoretical Chemistry (IPTC), University of Tuebingen, Auf der Morgenstelle 15, D-72076, Tuebingen, Germany; anna.staerz@ipc.uni-tuebingen.de (A.S.); inci.can@ipc.uni-tuebingen.de (I.B.); helena.brinkmann@student.uni-tuebingen.de (H.B.); sina.herrmann@ipc.uni-tuebingen.de (S.H.); benjamin.junker@ipc.uni-tuebingen.de (B.J.); upw@ipc.uni-tuebingen.de (U.W.)

^2^ European Synchrotron Radiation Facility (ESRF), 71 Avenue des Martyrs, 38043 Grenoble, France; david.degler@esrf.fr

^3^ Institut de Physique et Chimie des Matériaux de Strasbourg (IPCMS), UMR 7504 CNRS-Université de Strasbourg, 23 rue du Lœss, F-67034 Strasbourg cedex 2, France; mounib.bahri@ipcms.unistra.fr (M.B.); ovidiu.ersen@ipcms.unistra.fr (O.E.)

^4^ Institute of Catalysis Research and Technology (IKFT) and Institute for Chemical Technology and Polymer Chemistry (ITCP), Karlsruhe Institute of Technology, Kaiserstr. 12, 76131 Karlsruhe, Germany; dmitry.doronkin@kit.edu (D.E.D.); anna.zimina@kit.edu (A.Z.); grunwaldt@kit.edu (J.-D.G.)

***** Correspondence: nb@ipc.uni-tuebingen.de; Tel.: +49-(0)7071-29-78761

Table S1. Concentrations used for the loading preparations.

| **0.5 g of Base Material** | **M(RhCl_3_*H_2_O) [g]** | **Loading Level** |
| --- | --- | --- |
| **WO_3_** | 0.0113 g | 2.50 at.% |
|  | 0.0226 g | 5.00 at.% |
| **In_2_O_3_** | 0.0038 g | 0.50 at.% |
|  | 0.0207 g | 2.75 a.% |
| **SnO_2_** | 0.0035 g | 0.50 at.% |
|  | 0.0208 g | 3.00 at.% |


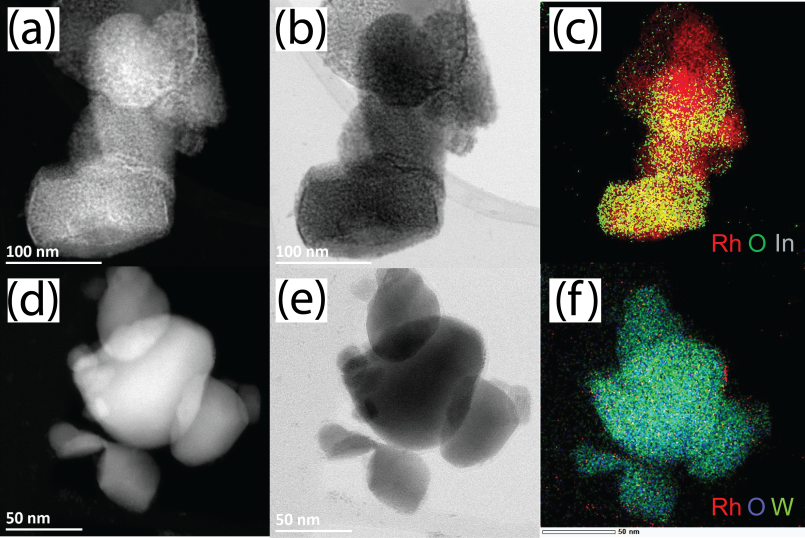


Figure S1. STEM images of 2.75 at.% Rh-Loaded In_2_O_3_. (a) STEM-HAADF (b) STEM-BF and EDS elemental mapping images (c), STEM images of 5 at.% Rh-Loaded WO_3_. (d) STEM-HAADF (e) STEM-BF and EDS elemental mapping images (f).


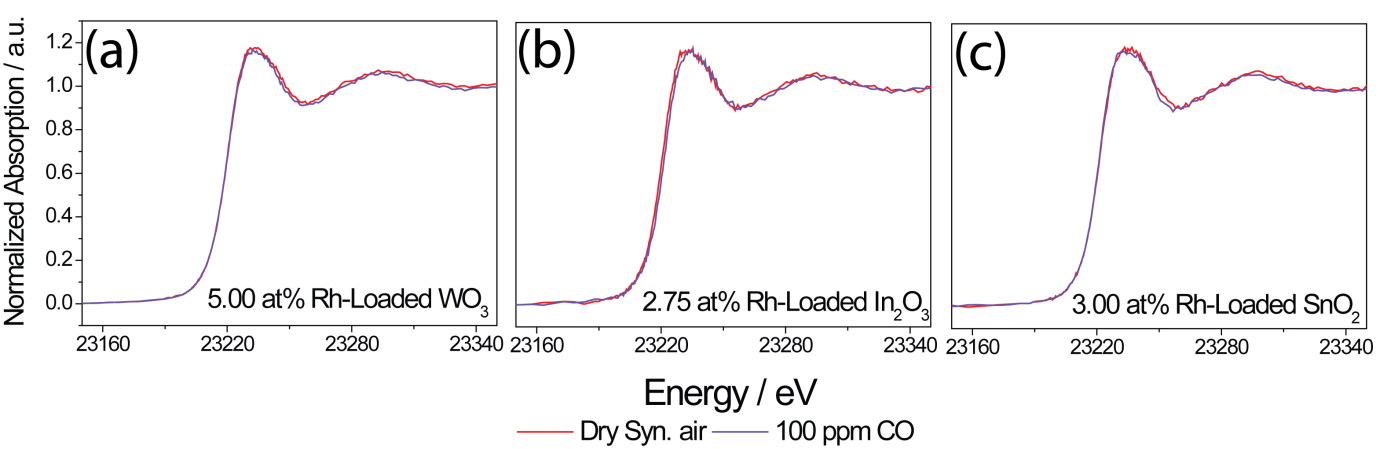


Figure S2. Rh K-edge XANES spectra of 5 at.% Rh-loaded WO_3_ (a), 2.75 at.% Rh-loaded In_2_O_3_ (b) and 3.00 at.% Rh-loaded SnO_2_ (c), recorded during different CO exposure in dry syn. air at 300 °C.
